# Equal Load-Carrying Design of Lapped Joints of Al–Cu Dissimilar Materials

**DOI:** 10.3390/ma13194293

**Published:** 2020-09-25

**Authors:** Zhihao Chen, Jianxiao Ma, Hongyuan Fang, Zhida Ni, Ping Wang

**Affiliations:** 1 School of Ocean Engineering, Harbin Institute of Technology, Weihai 264209, China; SunnyskyChen@outlook.com; 2 State Key Laboratory of Advanced Welding and Joining, Harbin Institute of Technology, Harbin 150001, China; hyfang@hit.edu.cn (H.F.); nzdhit@hotmail.com (Z.N.)

**Keywords:** equal load-carrying, lap joint, brazing, bearing design, fracture mode

## Abstract

In order to avoid the adverse effects of additional moment and stress concentration of traditional lap joints, a new lap joint was put forward, according to the concept of “equal load-carrying”. Through static analysis and brazing characteristics consideration, the equal load-carrying design method of Al–Cu lap joint based on brazing method was established. Through three types of brazing, the relationship among two fracture modes, brazing process and static tension curve of lap joint, was analyzed. The results demonstrated that the selection of solder was required to simultaneously meet the requirements of brazability and mechanical properties. A certain relationship existed between the fracture mode of the lap joint and the static tensile curve, while the segments of the static tensile curve corresponded to the fracture paths of the two fracture modes. When the brazing holding time was quite short, the interface bonding was poor, while the bearing capacity of the joint was low; when the holding time was suitable, the bearing capacity of the joint reached the corresponding highest, while the fracture mode conformed to the equal load-carrying design; when the brazing holding time was quite long, the bearing capacity of the joint remained at a high level, but the fracture mode was the same as the holding time was quite short.

## 1. Introduction

The static tensile load of the brazed joint is that “the static tensile bearing capacity of the brazed joint is not lower compared to the weakest base metal”, which could contribute to the overall capacity of the brazed structure [1]. When the traditional lap joint is subjected to axial tension, it will produce an additional bending moment at the joint position. Since the position of the lap joint is subjected to the tension-bending compound load, what we are concerned about in the service process is the bearing capacity of the brazed joint. Even if the final fracture location is the base metal, the bearing capacity of the joint will always be lower compared to the base metal strength. This type of joint does not fully utilize the inherent strength of the base metal. The thicker the plate, the higher the additional moment, whereas the more severe the bearing capacity decrease of the joint.

In the industrial field, aluminum and copper are good conductive materials. Aluminum has lower density and lower price than copper, but the corresponding conductivity is lower, while the strength is lower compared to copper. In order to fully utilize the excellent properties of copper and aluminum, it is necessary to join copper and aluminum to form a composite structure of copper and aluminum. Both copper and aluminum are easily oxidized metals, and their melting points and coefficients of thermal expansion are very different. Excessive chemical metallurgical reactions are prone to generate coarse brittle compounds, so it is difficult to connect with traditional fusion welding methods: welding deformation of pressure welding and explosive welding processes are very large and processes such as friction welding and diffusion welding are only suitable for simple components with regular shapes. Brazing has the advantages of small deformation and easy operation, which can meet the connection requirements of complex components [2]. Al–Cu dissimilar brazing is generally used in the socket connection of copper and aluminum pipes in the refrigeration industry, central air-conditioning copper and galvanized pipes, copper and aluminum terminals in the substation industry, copper and aluminum leads, and radiator tubes in the electronic and electrical industry. Brazing includes the selection of brazing material, brazing parameters (including current, voltage and speed), and preheating as well as different brazing process. Finally, the form of brazing joint will ultimately have different effects.

Regarding brazing dissimilar materials, Furuya, H.S. et al. [3] carried out dissimilar laser lap brazing formations between aluminum and pure copper by adding nickel, which highly improved the strength of Al–Cu dissimilar joints. Liu et al. [4] added rare earths (La and Nd) to Zn–10Al–5Cu solder and found that the brazed seam shear strength of the Cu–Al butt joint reached the maximum when the content of rare earth element was 0.15%. Ma et al. [5] proposed the surface sputtering Ni/Al double-layer aluminum foil brazing and heating brazing method. Regarding brazing parameters, Zhou et al. [6] analyzed the relationship between the shear strength of the joint in the Al (5052)–Cu (T2) brazing zone, where the joint’s strength reached the maximum 17.66 MPa when the current was 35 A. Li et al. [7] studied the effects of brazing speed on the microstructure and properties of Al–Cu dissimilar metal lap joints, and it was found that internal crack defects were significantly reduced and the sound lap joint could be obtained when the brazing speeding was 1000 mm/min. Regarding preheating, Qin et al. [8] studied the properties of Fe–Al dissimilar metal brazing joints under different preheating temperatures, and the bearing capacity of the joint was improved when the preheating temperature was 100 °C. Tan et al. [9] studied the effect of preheating on zinc solder on Al–Cu lap laser brazing, as preheating can increase the diffusion area of the filler metal and cause the growth of the reaction layer. The above-mentioned scholars have conducted a lot of systematic research on brazing technology. Regarding brazed joints, He et al. [10] utilized double aluminum hot wire brazing for dissimilar materials of aluminum alloys and stainless steels, for which, the effect of heat input on butt joints was studied, and it was found that with the increase in the brazing heat flow, the thickness of the IMC increased significantly, while the strength of the brazed joint decreased. Sing et al. [11] utilized a novel brazing process to produce a lap joint from aluminum and steel, and it was found that the strength of the brazed joint decreased with the increase in aluminum thickness. Chen et al. [12] prepared aluminum alloy linear joints with a closed square butt structure by modifying the cold metal transfer cycle step brazing process. Rheingans et al. [13] studied the relationship between microstructure and temperatures of copper, aluminum, and other materials at the joint, finally optimizing the joint design. Wei et al. [14] obtained Al/Cu lap joint by multi-pass friction stir brazing (FSW),The microstructure and bonding strength of the Al/Cu joint were analyzed, an overlap joint without defect inside the cross section was obtained when the stir pin interval was 3 mm and the plunge depth was 0.2 mm.

In the selection of solders, the brazability of the material, the diffusion, and interface reaction between the solder and the base metal has often been given attention, but less attention has been paid to the mechanical properties of the solder and the structural design of the joint. The brazed joints are generally divided into butt joints and lap joints, but each has certain advantages and disadvantages. Consequently, for research purposes, these joints were combined. In this work, an improved lap joint and the corresponding equal load-carrying design principle were proposed. Additionally, the Al and Cu lap joints were produced based on the brazing method. The possible fracture mode and mechanical properties of the joint under static tension were analyzed. Based on the test results of the three brazing processes, the relationship between fracture mode, mechanical properties, and joint quality was deduced.

## 2. Materials and Methods about the Design of Lap Joint

### 2.1. Equal Load-Carrying Lap Joint Form

**Figure 1:**
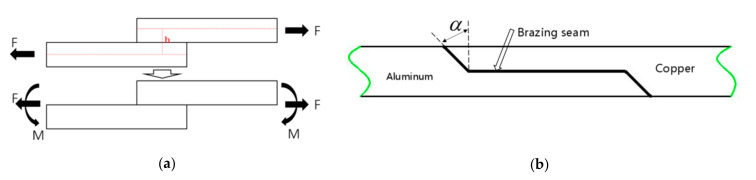
Traditional lap joint and improved lap joint. (**a**) Lap joint with addition bending moments. (**b**) Improved lap joint.

A lap joint is composed of two parts: overlapping joints with good force condition and low stress concentration, and large static load or dynamic load, which is one of the most common types of joints in brazing structures. As presented in Figure 1a, When the lap joint is subjected to a concentrated force F, the force is not collinear, and the distance of the line of action is h, so a bending moment will be generated, which can be decomposed into collinear force and bending moment M = F·h.

In order to reduce the structure weight, while additional bending moment and stress concentration were not produced, through which in turn, the process of mechanical analysis could be simplified and the requirements of equal load-carrying could be met, the traditional lap joint was improved to the joint form, as presented in Figure 1b.

### 2.2. Selection Principle of Solder

**Figure 2:**
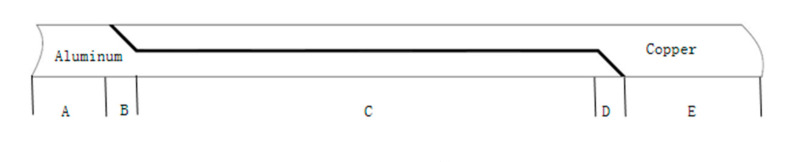
Five areas of lap joints.

Then equal load-carrying design comprised the performance parameters utilization of the base metal and the brazing filler metal as the initial parameters of joint design, while the brazed joint design method and criteria were utilized with the same load-bearing capacity as the base metal. The joint form and base metal are known in the case of circumstance, while the equal load-carrying design is the selection problem of the solder. The solder must not only meet the brazability of the material, but it is also required to have a certain tensile strength value. The following was derived from the tensile strength perspective. As presented in Figure 2, the lap joint was divided into five zones. The zone A was a pure material aluminum area, the zone E was a pure material copper area, zones B,C,D were mixed material area, and in terms of aluminum content, zone B > zone C > zone D. With the assumption that the tensile strength of copper was higher than that of aluminum, it could be deduced that for the pure material area, the strength of zone A was the lowest, whereas for the mixed material area, according to the ratio of regional cross-section materials, the strength sorting showed that the strength of zone B was the lowest.

**Figure 3:**
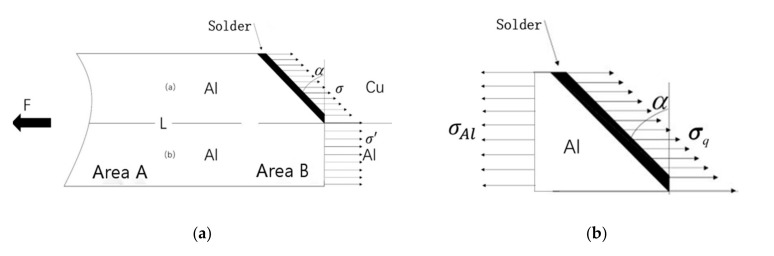
Stress analysis in area B. (**a**) Exposure of internal stress through cross-section method. (**b**) Schematic diagram of force analysis above L line.

Assuming that the stress on the section located perpendicularly to the loading direction was equal everywhere, as presented in Figure 3, when conducting the static tensile test, the tensile force is F, according to Newton’s third law, the force in area B is equal to that in F, and the direction is opposite, for which, only the part above the L line could be analyzed. Considering factors such as softening of base metal, internal defects within joint, diffusion, and interface reaction, the load-bearing requirements can be expressed through Equation (1). The meaning of the inequality sign needs to be explained. As shown in Figure 3b, in order to balance the stresses on the left and right sides, σ_Al_cosα ≤ σ_q_ can be obtained. However, due to the influence of the welding process, welding defects, the reduction of the interface strength of the welded intermetallic compound, and the softening of the base metal will inevitably occur. Obviously, these factors must also be taken into consideration. Therefore, in light of the above three points, three parameters were proposed and added to the left of the inequality sign to consider the impact of welding. Their interpretation and analysis are explained below in Equation (1). The meaning of the unequal sign on the right side is that the load-bearing capacity of the joint did not have to be higher compared to the strongest base metal.

μβh_0_σ_Al_cosα ≤ σ_q_ (1)

where σ_q_ is the tensile strength of the solder; σ_Al_ is the tensile strength of aluminum; μ is the brazing defects coefficient; β is the softening coefficient of the base metal, while h_0_ is the influence coefficient of diffusion and interfacial reaction.

Prior to brazing, it was necessary to select the appropriate brazing filler metal, in order to determine the parameters in Equation (1). Among the filler metals, 1060 pure aluminum [15] and H62 brass [16] were selected as the base materials, (both equipment’s and chemicals are from Hegang Shanghai Welding Co., LTD, Shanghai, China) while the corresponding chemical compositions are presented in Table 1 and Table 2 (equipment from Hegang Shanghai Welding Co., LTD, Shanghai, China), and the corresponding tensile strengths are presented in Table 3. The softening coefficient of base metal was related to the specific brazing process. Comparing the tensile strength of F state and O state aluminum (F state is a free state, that is, the state of the material before brazing, and O state is an annealed state), the softening coefficient of base metal was 0.556. The influence coefficient of brazing defects refers to the engineering regulations of brazing rate, and more than 75% was qualified. It was assumed that the effect of brazing rate on strength was fully reflected in the reduction of bearing section, while the maximum value of the brazing defect influence coefficient was 1.33, on the premise of meeting the requirement of the engineering brazing rate.

The above parameters were substituted into Equation (1) and h_0_ was conservatively estimated to be 2, so the strength of the solder would not be lower than 130 MPa. The tensile strength and melting point of the Zn-based solder Zn–5Al–3Cu [17] were 131 MPa and 390 °C, while the corresponding chemical composition are presented in Table 4 (both equipment’s and chemicals from Hegang Shanghai Welding Co., LTD, Shanghai, China), through which, the requirements of brazability and mechanical properties could be met at the same time.

## 3. Scratch Brazing of Specimen

**Figure 4:**
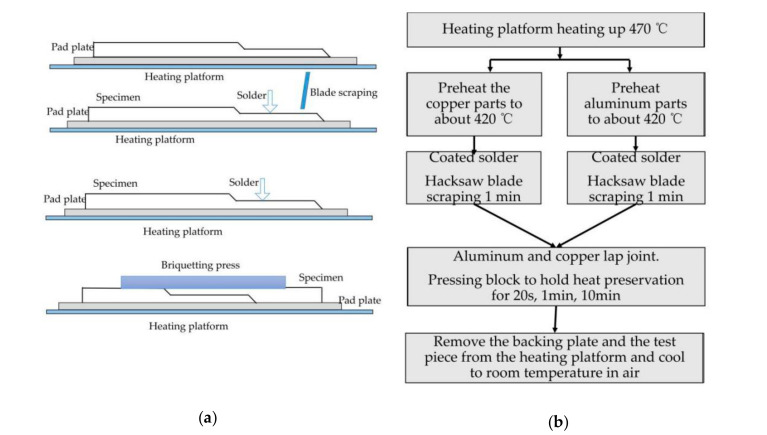
Brazing process parameters. (**a**) Schematic diagram of brazing process. (**b**) Brazing process.

The purpose of this section is to obtain usable brazing joints. This paper does not intend to find the optimal welding process parameters, but focuses on the previous joint design [18]. In the experiment, scratch brazing was utilized to connect 1060 pure aluminum and H62 brass. The corresponding technological process is presented in Figure 4. The purpose of scraping was to effectively remove the oxide film from the material surface, while the effect of scraping was better than that of the brazing flux. Subsequent to scraping, the heat preservation time was set to 20 s, 1 min, and 10 min, respectively, as a comparative experiment, the mass of the briquettes was 200 g.

**Figure 5:**
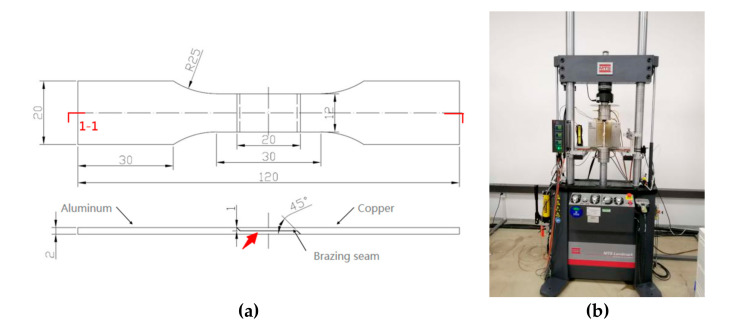
Size of tension test specimen and experiment apparatus. (**a**) Size of tension test specimen. (**b**) Experiment apparatus MTS.

The dimensions of the stretched parts followed the requirements of the American Welding Society (AWS) design handbook for calculating fillet braze sizes. The thickness of the plate was 2 mm (such a thin plate requires very high welding requirements when brazing. In this case, some unqualified weldments will appear, so the thickness of the material should be at least 2 mm or more), the lap length of the joint was 20 mm, and the bevel angle was 45°, as presented in Figure 5. The preparation of the sample was completed by wire cutting including the overall outline and lap interface. The sampling position of the Scanning Electron Microscope (SEM) is marked in Figure 5a (The equipment was from Opton, Ismaning, Germany). The tensile test was carried out by Mechanical Testing Simulation (MTS), the experiment apparatus is presented in Figure 5b (The equipment was from Sinotest Equipment Co., Ltd.; Beijing; China). Subsequently, the samples were treated with sandpaper of 200#, 500#, 800#, and then polished with diamond of 1 micron.

## 4. Result Analysis and Discussion

### 4.1. Microstructure of Brazing Seam and Interface Layer

**Figure 6:**
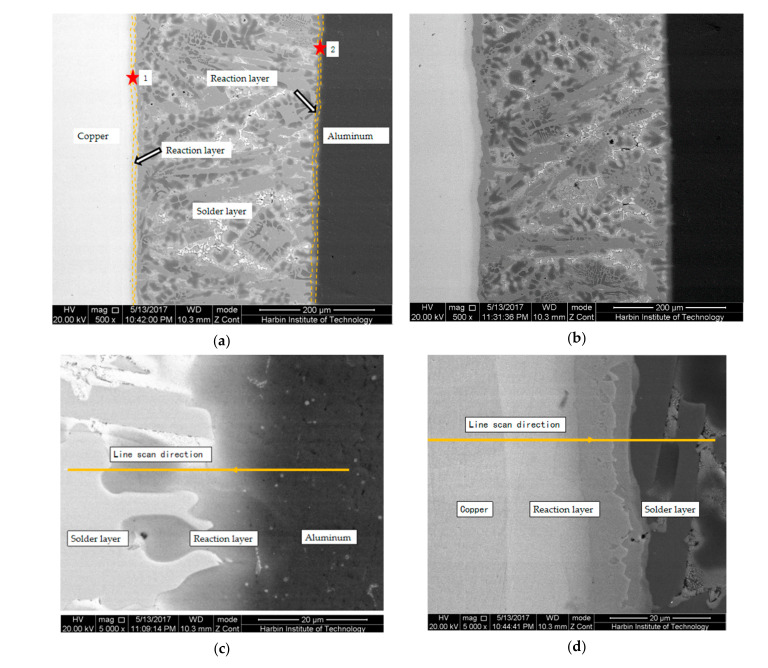
SEM photo of solder seam. (**a**) SEM photo of brazing seam on both sides of copper and aluminum held for 10 min. (**b**) SEM photo of brazing seam on both sides of copper and aluminum held for 1 min. (**c**) SEM photo of aluminum side brazing seam (point 2 as marked in Figure 6a). (**d**) SEM photo of copper side brazing seam (point 1 as marked in Figure 6a).

**Figure 7:**
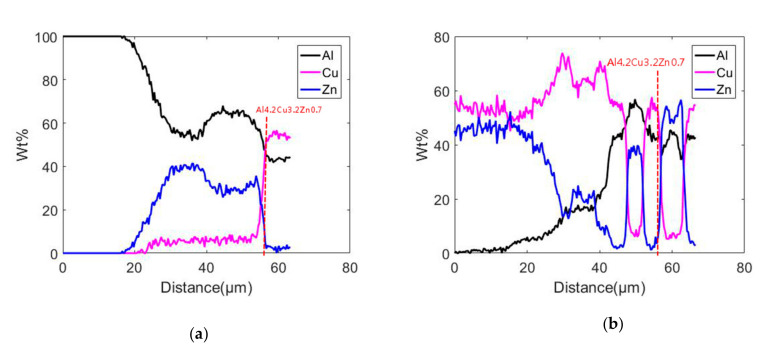
Results of line scanning on copper and aluminum sides. (**a**) Line scan of Al side. (**b**) Line scan of Cu side.

Figure 6 presents the microstructure of the brazing seam and the interface layer with the holding times of 1 min and 10 min, in which, the thickness of the reaction layer on the copper side were 8 mm and 13 μm, while the thickness of the reaction layer on the aluminum side were 16 mm and 21 μm, respectively. Overall, their interfaces were similar [19]. Due to the longer holding time, the thickness of the reaction layer was also relatively high. Here, we mainly analyzed the microstructure of the brazing seam and the interface layer with a holding time of 10 min. Figure 6b is a SEM photograph of the brazing seam on both sides of the copper and aluminum for 1 min. Figure 7a presents the line scanning results of points 1 and 2, which demonstrated that the base metal and solder had been metallurgically combined, while the selection of solder met the requirements of material brazability. It should be noted that the concept of Cu in Figure 7 is a chemical element, while copper used elsewhere in the paper is an alloy.

According to Figure 6c and Figure 7a, it can be seen that on the side of the base material aluminum, the zinc solder dissolved into the base material aluminum, resulting in a decrease in the weight percentage content of aluminum and an increase in zinc content during the line scan. The scanning distance was close to 60 μm (about 55 μm). When it was a micrometer, the intermetallic compound Al_4.2_Cu_3.2_Zn_0.7_ was formed. According to Figure 6d and Figure 7b, when the line scan was performed on the copper side of the base material, the content of aluminum was small at the beginning. As the line scan moved, the dissolution of aluminum and copper into the joint brazing layer will cause aluminum in the brazing layer. As the copper atom content increased, the zinc atom content decreased, then began to fluctuate. When the line scan movement distance was about 56 microns, the intermetallic compound Al_4.2_Cu_3.2_Zn_0.7_ was formed [20].

### 4.2. Fracture Mode and Mechanical Properties

**Figure 8:**
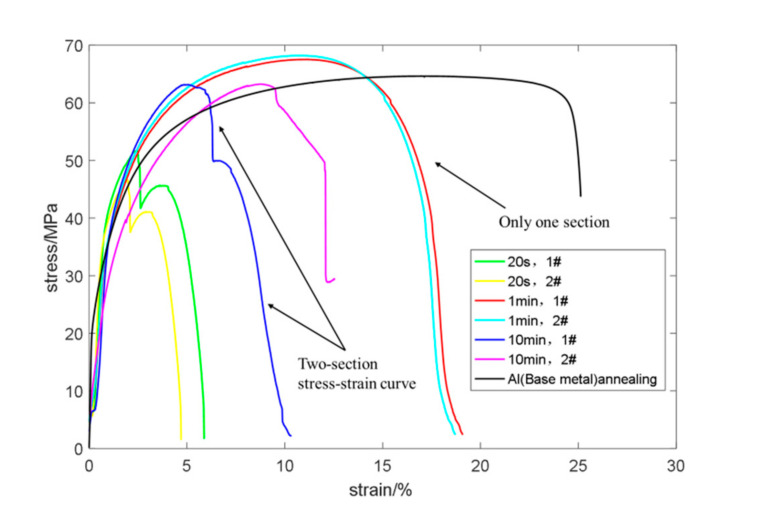
Tensile curves of tension test specimens under different brazing processes.

Figure 8 shows two types of stress–strain curves. The stress–strain curve of most of the curves presented two sections. For example, in the case of 20 s and 10 min, the stress–strain curve of 1# and 2# was not a smooth curve, but a turning curve. It was composed of two parts: the curve before turning and the curve after turning, indicating that these two parts were caused by different expansion paths during fracture, which was called mode Ⅰ. When the holding time was 1 min, the stress-strain curve of 1# and 2# was a smooth curve, indicating that the specimen fracture was caused by an expansion path, which was called mode Ⅱ. The related introduction of mode I and mode II is in the following part of this article.

**Figure 9:**
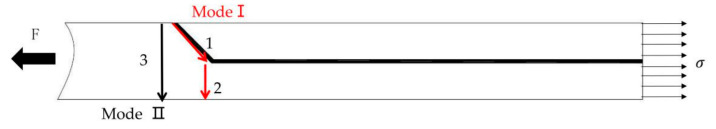
Two fracture modes of lap joint.

The following explains how the two modes correspond to the direction of crack propagations. As presented in Figure 9, the failure path of fracture mode Ⅰ was 1→2 and that of fracture mode Ⅱ was 3, in which, mode Ⅰ was caused by the selection of solder or unreasonable brazing technology. Mode Ⅱ was fractured upon the base metal, which was the ultimate goal (fracture occurs on the base material first) of the equal load-carrying design.

**Figure 10:**
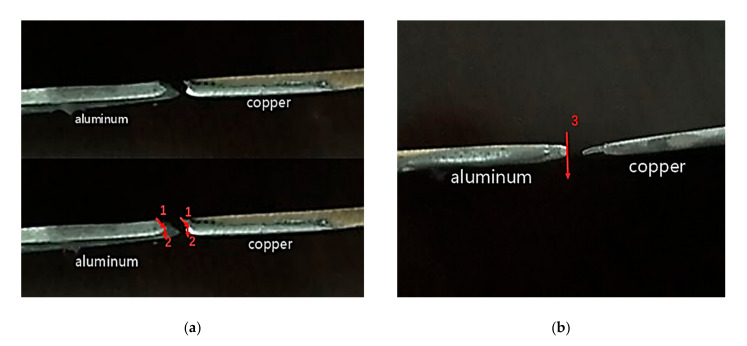
Macro morphology under different fracture modes. (**a**) Macro-morphology of mode Ⅰ fracture. (**b**) Macro-morphology of mode Ⅱ fracture.

In mode Ⅰ and at the intersection of the two segments, the material of path 1 was completely fractured, while the macroscopic morphology of the two fracture modes is presented in Figure 10. According to the bearing capacity of the samples, the curves in the diagram could be divided into two categories from which the tensile strength of the two specimens insulated for 20 s was only 52 MPa, whereas the tensile strengths of the four samples with holding times of 1 min and 10 min exceeded 63 MPa, reaching up to 67 MPa. Among the above six samples, the elongation of the two samples with heat preservation of 1 min was higher, while the elongation of the other samples was lower. Moreover, the elongations of the two samples with heat preservation of 10 min varied highly, which were 10% and 13%, respectively, which shows that the welding quality was unstable.

As seen in Figure 10, we obtained the macro morphology under different fracture modes through high-definition cameras. We can clearly see two fracture modes from Figure 10a,b, which verifies the two modes illustrated in Figure 9.

Table 5 summarizes that the thickness of reaction layer influences the coefficient of diffusion, and interfacial reaction strength influences the coefficient, tensile strength, and fracture path under different holding times.

When the holding time was 20 s, the diffusion and metallurgical reaction of the interface had not been fully completed, the reaction layers were thinner, resulting in poor interfacial bonding, while the influence coefficient h_0_ was below 2. When the joint was subjected to the tensile stress of 52 MPa, the position of the brazing joint began to fracture. Consequently, the strength was low and the elongation was quite low.

It should be noted that the parameter *h*_0_ represents the strength change of the solder before and after brazing (i.e., the strength of the solder before brazing was 131 MPa, but after welding, the strength was reduced due to melting and solidification and intermetallic compounds). Therefore, the tensile strength of the specimen at 10 min was taken as the strength of the solder after brazing, because it was long enough in time. So, when the holding time was 10 min, $h_{0}=\frac{131}{63}=2.08$, but when the holding time was 20 s, due to incomplete metallurgical reaction, h_0_ was still less than 2.

When the holding time was 1 min, the diffusion and metallurgical reaction of the interface could be fully completed, while the reaction layer was thinner. At the later stage of tensile testing, when aluminum entered the stage of plastic deformation, the thinner reaction layer could withstand a certain amount of plastic deformation when the specimens were stretched, during which, the brittle fracture would not occur within the reaction layer, whereas the influence coefficient exceeded 2.5. The bearing capacity of the joint was equal to the tensile strength of the base metal, while the joint fractured according to mode Ⅱ.

When the holding time was 10 min, the diffusion and metallurgical reaction of the interface could be fully completed. Since the holding time was quite long, the reaction layer on the interface was thick enough that it could reach 20 μm. At the later stage of tensile testing, aluminum entered the stage of plastic deformation, but the reaction layer was brittle and could not synchronize with the plastic deformation of aluminum, which led to the early fracture of the joint. The influence coefficient was 2.08. Consequently, the bearing capacity of the joint was maintained at a high level, reaching 63 MPa. However, the elongation had decreased to a certain extent.

**Figure 11:**
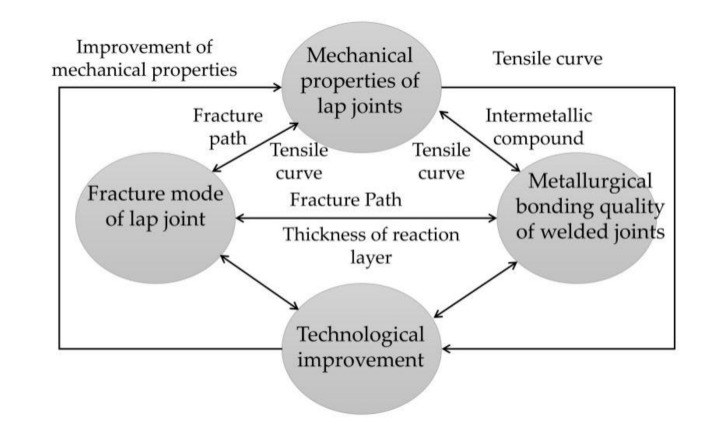
Schematic diagram of the relationship among the properties of thee lap joint, metallurgical combined quality, fracture modes, and brazing processes.

Figure 11 presents the relationship between the brazing process [21], mechanical properties, fracture mode, and bonding quality of the lap joint, through which the metallurgical bonding quality determined the fracture mode, while the thickness of the reaction layer was the key factor. If the diffusion time was short, the interface bonding would be poor, whereas if the diffusion time was too long, the interface reaction layer would be brittle, while the fracture mode was mode Ⅰ. Therefore, the fracture mode was utilized to actualize the brazing process improvement. Finally, the load-bearing capacity of the lap joint was improved and the equal load-carrying design was realized.

## 5. Conclusions

In this paper, a new type of lap joint was designed. Through a reasonable design, its strength can be the same as the strength of the base material, and no additional bending moment will be generated, which is good for the service of brazed joints. Through the analysis of its stress–strain curve, two crack propagation modes were found, which are unique to this joint. From the above analysis, the following points can be summarized:

(1) On the basis of the form determination of a new lap joint, combined with the brazing of an Al–Cu dissimilar metal, according to the characteristics of static analysis and brazing, the design method of static tension and loads was given.
(2) Two fracture modes of lap joints were analyzed from which mode Ⅱ was the ultimate goal of equal load-carrying design, and mode Ⅰ might be due to the wrong selection of solder or unreasonable brazing process.
(3) The relationship between fracture mode, static tensile curve and brazing quality was deduced. The results demonstrated that the static tensile curve of mode Ⅰ followed a two-stage rule, while mode Ⅱ presented a one-stage law. When the brazing holding time was too long or too short, the fracture mode of the joint was mode Ⅰ.

## Figures and Tables

**Table 1:** Chemical composition of 1060 pure Al (mass fraction/%).

Si	Cu	Mg	Zn	Mn	Cr	Fe	Al
0.25	0.1	2.2–2.8	0.1	0.1	0.15–0.35	0.4	Bal.

**Table 2:** Chemical composition of H62 brass (mass fraction/%).

Cu	P	Bi	Sb	Pb	Fe	Zn
60.5–63.5	≤0.01	≤0.002	≤0.005	≤0.08	≤0.15	Bal.

**Table 3:** Tensile strength of base material.

Materials	Tensile Strength (MPa)
1060 Pure aluminum (F state)	112
1060 Pure aluminum (O state)	64
H62 Brass	417

**Table 4:** Chemical composition of Zn–5Al–3Cu (mass fraction/%).

Zn	Al	Cu	Mg	Si	Ag	Ni
84.5	7.10	4.5	0.84	0.24	1.76	1.05

**Table 5:** Different mechanical parameters and fracture modes of the tension testing specimen under different brazing processes.

Brazing Holding Time	20 s	1 min	10 min
Thickness of reaction layer on aluminum side (μm)	<2	7	20
*h*_0_	<2	>2.5	2.08
Tensile strength (MPa)	52	67	63
Fracture path	1→2	3	1→2
Fracture mode	Mode Ⅰ	Mode Ⅱ	Mode Ⅰ
Elongation	5–6%	19%	10–13%
